# Key findings from the UKCCMP cohort of 877 patients with haematological malignancy and COVID‐19: disease control as an important factor relative to recent chemotherapy or anti‐CD20 therapy

**DOI:** 10.1111/bjh.17937

**Published:** 2021-11-10

**Authors:** Stephen Booth, Helen M. Curley, Csilla Varnai, Roland Arnold, Lennard Y. W. Lee, Naomi A. Campton, Gordon Cook, Karin Purshouse, James Aries, Andrew Innes, Lucy B. Cook, Oliver Tomkins, Helen S. Oram, Michael Tilby, Austin Kulasekararaj, David Wrench, Saoirse Dolly, Tom Newsom‐Davies, Ruth Pettengell, Abigail Gault, Sam Moody, Sajjan Mittal, Mohammed Altohami, Tania Tillet, Jack Illingworth, Leena Mukherjee, Jane Apperly, John Ashcroft, Neil Rabin, Jonathan Carmichael, Jean‐Baptiste Cazier, Rachel Kerr, Gary Middleton, Graham P. Collins, Claire Palles, UKCCMP team

**Affiliations:** ^1^ Oxford NIHR Biomedical Research Centre Department of Haematology Churchill Hospital Oxford UK; ^2^ Institute of Cancer and Genomic Sciences University of Birmingham Birmingham UK; ^3^ Centre for Computational Biology University of Birmingham Birmingham UK; ^4^ Department of Oncology Oxford University Oxford UK; ^5^ Institute of Translational Medicine Birmingham Health Partners Birmingham UK; ^6^ NIHR (Leeds) MIC, Leeds St James's Teaching Hospital, University of Leeds Leeds UK; ^7^ Edinburgh Cancer Research Centre University of Edinburgh Edinburgh UK; ^8^ Barts Health NHS Trust London UK; ^9^ Imperial College Healthcare NHS Trust London UK; ^10^ Leswisham and Greenwich NHS Trust London UK; ^11^ Queen Elizabeth Hospital Birmingham UK; ^12^ Department of Haematology King's College Hospital London UK; ^13^ Guys and St Thomas' NHS Foundation Trust London UK; ^14^ Chelsea and Westminster Hospital NHS Foundation Trust London UK; ^15^ St Georges University Hospitals NHS Foundation Trust London UK; ^16^ NCCC Northern Centre for Cancer Care The Newcastle Upon Tyne NHS Foundation Trust Newcastle UK; ^17^ Northampton General Hospital NHS Trust Northampton UK; ^18^ Royal United Hospitals Bath Bath UK; ^19^ Barking Havering and Redbridge University Hospitals NHS Trust Essex UK; ^20^ Beatson West of Scotland Cancer Centre Glasgow UK; ^21^ Mid Yorkshire Hospitals NHS Trust Wakefield UK; ^22^ University College London Hospitals London UK; ^23^ Institute of Immunology and Immunotherapy University of Birmingham Edgbaston Birmingham UK

**Keywords:** COVID‐19, haematological malignancies, cancer treatments

## Abstract

Patients with haematological malignancies have a high risk of severe infection and death from SARS‐CoV‐2. In this prospective observational study, we investigated the impact of cancer type, disease activity, and treatment in 877 unvaccinated UK patients with SARS‐CoV‐2 infection and active haematological cancer. The primary end‐point was all‐cause mortality. In a multivariate analysis adjusted for age, sex and comorbidities, the highest mortality was in patients with acute leukaemia [odds ratio (OR) = 1·73, 95% confidence interval (CI) 1·1–2·72, *P* = 0·017] and myeloma (OR 1·3, 95% CI 0·96–1·76, *P* = 0·08). Having uncontrolled cancer (newly diagnosed awaiting treatment as well as relapsed or progressive disease) was associated with increased mortality risk (OR = 2·45, 95% CI 1·09–5·5, *P* = 0·03), as was receiving second or beyond line of treatment (OR = 1·7, 95% CI 1·08–2·67, *P* = 0·023). We found no association between recent cytotoxic chemotherapy or anti‐CD19/anti‐CD20 treatment and increased risk of death within the limitations of the cohort size. Therefore, disease control is an important factor predicting mortality in the context of SARS‐CoV‐2 infection alongside the possible risks of therapies such as cytotoxic treatment or anti‐CD19/anti‐CD20 treatments.

## Introduction

Patients with haematological malignancies have been shown to be at higher risk of severe infection and death from SARS‐CoV‐2 infection compared to the general population or patients with solid‐organ malignancies in both large case series and population data.^1^, ^2^, ^3^, ^4^ A meta‐analysis of 3377 patients with haematological malignancies and SARS‐CoV‐2 infection reported a 34% risk of death in cases diagnosed during the first eight months of the pandemic, with particularly high risk in patients with acute leukaemia.^5^ High mortality has also been reported in patients with multiple myeloma (MM)^6^ and chronic lymphocytic leukaemia (CLL).^7^ Clinical practice guidelines have therefore recommended prioritisation of cancer treatments which are less immunosuppressive, require less hospital attendance, and have the highest likelihood of benefit.^8^

The impact of anti‐cancer treatments on mortality in SARS‐CoV‐2‐positive haematological patients has been investigated in increasingly large cohorts, although with variation in methodology and reported findings. Two large US studies reported an association between cytotoxic chemotherapy in either the past month or the past three months and increased mortality,^9^, ^10^ although these cohorts included patients with historic diagnoses of cancer, which limits interpretation. Three further studies, each with >600 patients with haematological malignancies and including a meta‐analysis of 13 studies, observed no statistically significant association between chemotherapy administered in the 28 days before SARS‐CoV‐2 infection and mortality.^3^ ^,^ ^5^ ^,^ ^11^ The effect of some specific drug classes has been investigated in smaller cohorts (<150 patients), again with variation in findings, as typified by the effect of recent anti‐CD20 therapy in lymphoid disease.^12^, ^13^ Variation between patients with different haematological malignancies has not been defined.

The UK Coronavirus Cancer Monitoring Project (UKCCMP) was launched on 18 March 2020, as a UK‐wide cancer reporting network for cases of COVID‐19 in patients with cancer.^14^ Here we report on 877 non‐overlapping UK patients with haematological cancers, registered to either UKCCMP or to the UK Myeloma Forum prospective clinical audit.^15^ We provide information on the impact of haematological disease subtype, line of treatment, treatment intent and specific cancer treatments on all‐cause mortality and COVID‐19‐specific mortality. Importantly the data suggest that poorly controlled disease represents a significant risk for poor COVID outcomes above that posed by chemotherapy or anti‐CD20 agents used to treat the disease. We also present information on the COVID‐19 treatments given to haematological cancer patients in the UK. Comparison of UKCCMP data to data from the Office for National Statistics^16^ and the Haematological Malignancy Research Network^17^ demonstrates the higher risk of hospitalisation following SARS‐CoV2 infection in patients with haematological cancers.

## Methods

### Data collection and study design

Patient data were collected as per Lee *et al*.^1^ and Varnai *et al*.^3^ for the UKCCMP. In response to the results of analysis of this cohort, the myeloma cohort was expanded by collaboration with the UK Myeloma Forum^15^ (UKMF), a contemporaneous clinical registry, in order to improve the power of analysis of treatment types in myeloma. For UKCCMP, 54 centres entered anonymised data to REDCap electronic data capture tools,^18^, ^19^ hosted at the Institute of Translational Medicine, University of Birmingham. Guy’s and St Thomas’ (*n* = 44) and King’s College Hospital NHS Foundation Trusts (*n* = 33) collected data using their own proformas.^20^ Haematological cancer patients with a positive SARS‐CoV‐2 test result and an active cancer diagnosis between 3 March 2020 and 10 May 2021 and registered on the REDCap system before 9 June 2021 were included. Active haematological cancer was defined as any untreated cancer or cancer treated palliatively at any time point, any patient on active cancer treatment, and those treated within the past 12 months with either systemic anti‐cancer therapies (SACT) and/or radiotherapy. SARS‐CoV‐2 was confirmed by reverse transcription polymerase chain reaction (RT‐PCR) in 97% of cases (*n* = 981/1011). Teenage and young adults (<25 years) with haematological cancers and those with presumed second primary cancers were excluded from the main analysis. Active treatment was defined by receipt within four weeks of the SARS‐CoV‐2 test. Treatment categories were: cytotoxic chemotherapy, steroid, targeted therapies (small‐molecule inhibitors and monoclonal antibodies) immunotherapy (checkpoint inhibitors) and immunomodulatory. Detailed classification is presented in Table SI. We also captured stem cell transplants received up to one year prior and B‐cell‐depleting (anti‐CD20 or anti‐CD19, excluding CAR‐T cell therapy), or T‐cell‐depleting (purine analogues or anti‐CD52) therapy received up to two years prior to the positive SARS‐CoV‐2 test.

### Data processing

Data was processed as per Varnai *et al*.^3^ Key comorbidities were defined as chronic kidney disease, chronic obstructive pulmonary disease, cardiovascular disease, diabetes, hypertension, or vascular disease. These comorbidities had previously been highlighted as associated with increased risk from SARS‐CoV‐2 infection and worse outcomes in the general population. Obesity was not included as a key comorbidity because body mass index (BMI) was missing for 431 patients. Patients were scored for the presence or absence of a key comorbidity and this variable was included in multivariate analyses. Haematological disease status was defined as one of: no treatment and asymptomatic (i.e. treatment‐naive patients on active surveillance), no treatment yet but indication to treat now, treated with ongoing response, treated and in complete remission or treated but relapsed/progressive disease. Line of treatment was analysed comparing three groups: untreated, first line and second and beyond. Wave 1 UK COVID‐19 infections were defined as those diagnosed before 1 August 2020. In order to investigate the impact of the government advice to shield, outcomes in those diagnosed before 30 March 2020 were compared to those diagnosed later.

### Statistical analysis

After the data curation process (described previously in Varnai *et al*.^3^) 877 patients with complete data for key multivariate analysis variables (age, sex and comorbidity status) and confirmed infection by RT‐PCR were available from UKCCMP and UKMF data (Fig S1) and were used for all analyses unless otherwise stated. All‐cause mortality was the primary outcome of interest, patients who died of any reported cause (COVID‐19 or non‐COVID‐19 causes) were compared to those who survived during the study time frame following a diagnosis of COVID‐19. A secondary outcome was COVID‐19‐specific death where COVID‐19 was listed as a significant contributing factor. For deaths to be included in this outcome variable COVID‐19 had to be listed on the death certificate as directly contributing to the death. For all‐cause death the mean follow‐up period for alive patients was 24 days and 12 days for deceased patients. For COVID‐specific death the mean follow‐up period for alive patients was 24 days and 11 days for deceased patients. When comparing the effect of a given cancer treatment on the outcome, all patients not on that treatment were the comparative group. The cancer subtypes were grouped into the following categories: (i) ALL (acute lymphoblastic leukaemia), AML (acute myeloid leukaemia) and MDS (myelodysplastic syndrome); (ii) MM (multiple myeloma) and plasmacytoma; (iii) CLL; (iv) CML (chronic myeloid leukaemia) and MPN (myeloproliferative neoplasms); (v) lymphoma, WM (Waldenström macroglobulinaemia), and other mature lymphoid leukaemia; and (vi) other/unspecified haematological malignancies. Definitions of treatment classifications are shown in Table II.

All data processing, statistical analysis and visualisation were performed in R (v.3.6.3; R Foundation for Statistical Computing, Vienna, Austria, https://cran.r‐project.org). Multivariable logistic regression was used to estimate odds ratios (OR) and 95% confidence intervals (CI) of each factor, adjusting for age, sex and comorbidity status. Additional covariates such as line of treatment and cancer type were included where indicated to explore their impact alongside the test variable. Patients with either no information or missing relevant data were excluded from the corresponding regression analyses.

Laboratory data for C‐reactive protein (CRP), lymphocytes and neutrophils were tested as binomial variables; high CRP (greater than the median CRP level for those input data), high lymphocytes (>5 × 10^9^/l), low lymphocytes (<1 × 10^9^/l), high neutrophils (>8 × 10^9^/l), low neutrophils (<2 × 10^9^/l). Multivariate logistic regression was used to estimate OR and 95% CI of each factor after adjustment for age, sex and comorbidity status.

For Office for National Statistics data, registrations of newly diagnosed cases of cancer in England during 2017 were used. All haematology was the sum of the individual cancer types ICD10: C81‐C96 and the other syndromes of interest D45 and D46 for those >25 years. The denominator for all tests was the overall value of Office for National Statistics registrations >25 years. For Haematological Malignancy Research Network data, incidence data were used for all those diagnosed >25 years: data were accessed on 10 March 2021. Two‐sided Fisher's exact tests were used to compare Haematological Malignancy Research Network proportions and Office for National Statistics proportions with corresponding UKCCMP proportions.

## Results

### Patient characteristics

From 896 patients reported to the UKCCMP and 115 patients reported to the UK Myeloma Forum, 877 patients were included as shown in Fig S1. Reasons for exclusion included lack of PCR confirmation, age less than 25 years, missing key variables and multiple active cancers. The characteristics of these 877 patients are shown in Table I. All‐cause mortality was 44% following COVID‐19 infection in the study follow‐up period. Increasing age and presence of multiple comorbidities were associated with higher overall mortality risk, but male sex was not (Table SII). Considering comorbidities separately, only cardiovascular disease and hypertension were significantly associated with higher risk of all‐cause death (Table SIII). Obesity (BMI > 30; Table SIII) and Black, Asian and Minority Ethnicity (BAME) (Table SII) were not associated with all‐cause mortality. High volumes of missing data for obesity and ethnicity resulted in their exclusion from adjustment in multivariate analyses.

**Table I:** The impact of line of treatment and haematological disease status on all‐cause death following diagnosis of COVID‐19.

	Total	Number of all‐cause deaths	OR (95% CI)	*P* value
No treatment	146	64 (44%)		
First line	304	116 (38%)	1·15 (0·75,1·77)	0·52
Second line and beyond	194	97 (50%)	1·68 (1·07,2·64)	0·025
Never treated, asymptomatic	60	19 (32%)		
Never treated, indication to treat now	50	25 (50%)	2·45 (1·09,5·5)	0·03
Treated with ongoing complete response or complete morphological remission	89	17 (19%)	0·84 (0·38,1·86)	0·67
Treated with some ongoing response	114	46 (40%)	1·97 (0·99,3·95)	0·055
Treated but with stable or progressive disease, relapse or not yet assessed	214	107 (50%)	3·21 (1·68,6·14)	<0·001

Age, sex and comorbidity status were included in the general linear model as covariates. Overall *P*‐value of line of treatment *P* = 0·049. Overall *P* value for impact of haematology disease status *P* < 0·001.

Altogether, 743 patients in this cohort were diagnosed with COVID‐19 in the UK’s first wave of COVID‐19, whereas 134 patients were diagnosed later. Date of COVID‐19 diagnosis was not significantly associated with risk of all‐cause mortality [OR = 0·9987 (95% CI 0·9972–1·0003), *P* = 0·115]. There was no significant increased risk of all‐cause mortality when those diagnosed before shielding advice (*n* = 268, of which 47% died) were compared to those diagnosed afterwards (*n* = 609, of which 23% died) [OR = 1·21 (95% CI 0·89–1·63), *P* = 0·219].

In total 91 (10%) patients were admitted to the intensive‐care unit (ITU), significantly more than amongst UKCCMP solid‐organ cancer patients (4%), an effect persistent in a multivariate analysis adjusted for age, sex and comorbidity status [*P* < 0·001, OR = 2·67 (95% CI = 1·92–3·71)]. The mortality rate for patients admitted to ITU was high at 68%.

### Impact of cancer subtype

**Fig 1:**
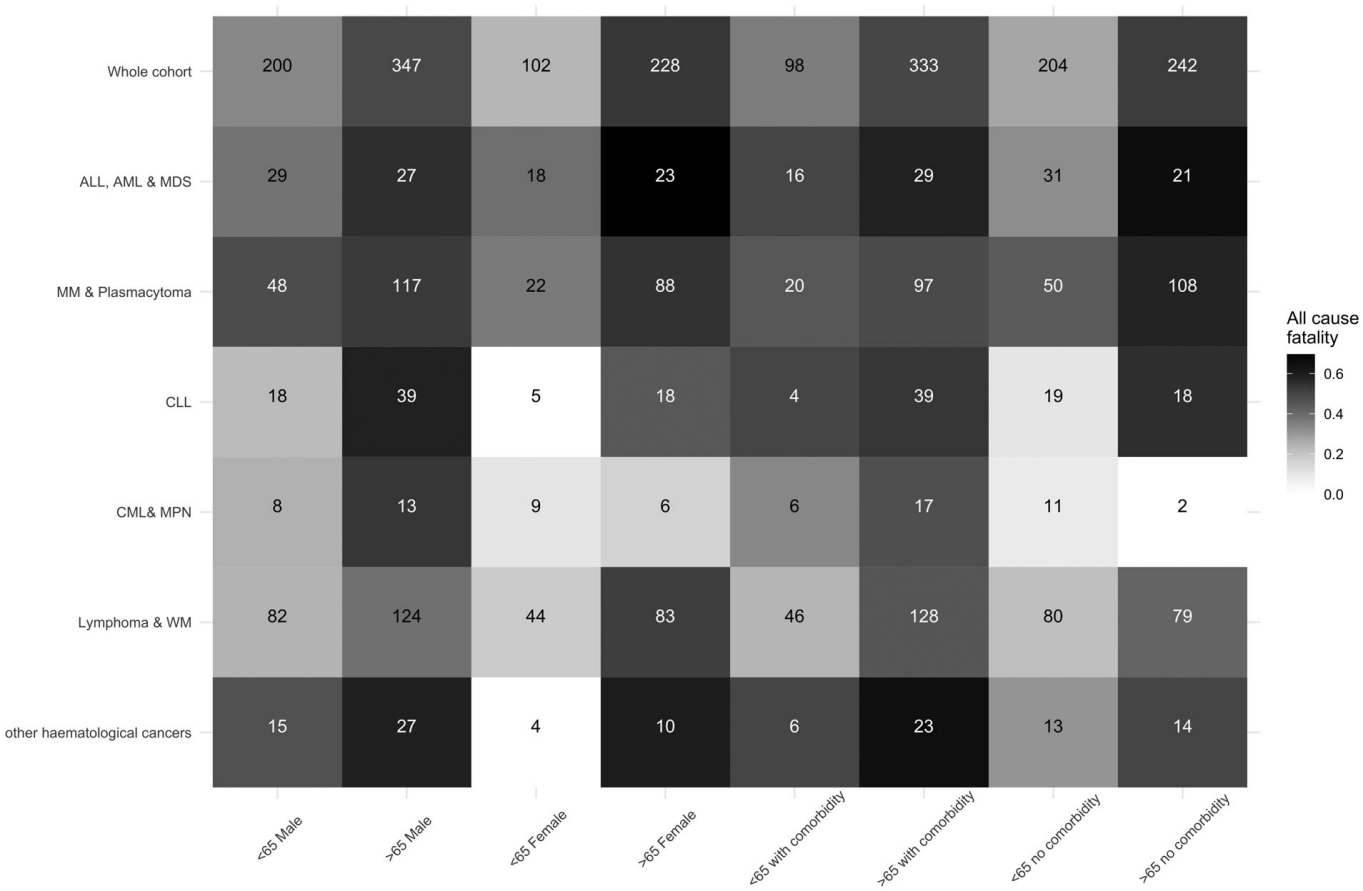
The Impact of age, sex and comorbidity on all‐cause fatalities split by haematological subgroup. All‐cause fatality is presented split by age (cut point 65), sex and presence or absence of a key comorbidity (chronic kidney disease, chronic obstructive pulmonary disease, cardiovascular disease, diabetes, hypertension, or vascular disease). Darker boxes signify higher fatality rates. The number represents all patients that fit into the two criteria (haematological subtype and characteristic). In all, 877 haematology cases were included. Overall, 44% died of all causes in the study period. Median age was 71, range 25–98. Age >65 *versus* aged <65 was significantly associated with increased all‐cause death [*P* < 0·001, odds ratio (OR) = 2·41, 95% confidence interval (CI) = 1·79–3·24]. Male sex was not significantly associated with all‐cause death (*P* = 0·832, OR = 0·97, 95% CI = 0·74–1·28). 431 out of 877 patients (49%) had one or more key comorbidities. Across all cases 52% of those with a key comorbidity died in the study period. Comorbidity status as a variable (0v1v2v3v4v5 or more comorbidities) was significantly associated with all‐cause death (*P* = 0·006, OR = 1·21, 95% CI = 1·06–1·4).

Haematological cancer subtype was associated with risk of mortality (Table SIV). The ALL, AML and MDS subgroups were significantly associated with increased risk of all‐cause death (Table I), whilst the lymphoma and WM subgroup were significantly associated with a lower risk of all‐cause death. The worst fatality rates were observed in ALL, AML and MDS patients who were over 65 years old and female, ALL, AML and MDS patients over 65 years old with no comorbidities and CML and MPN patients who were over 65 years old and male (Fig 1). An analysis of risk of mortality in patients with solid and haematological malignancies reported to UKCCMP identified that patients with ALL, AML and MDS had a statistically significant increased risk of all‐cause mortality compared to patients with digestive‐organ cancers in all analyses performed. Patients with myeloma and plasmacytoma were also highlighted as having a high risk of all‐cause death but this association was only significant in unadjusted analysis.^3^ Using the larger set of haematological cancer patients reported here we confirm that patients with any of the cancer types mentioned above have an increased risk of all‐cause mortality. The results were statistically significant for both AML, ALL and MDS patients [OR 2·6 (95% CI 1·6–4·2), *P* < 0·001] and patients with myeloma and plasmacytoma [OR 1·91 (95% CI 1·39–2·62), *P* < 0·001]. Patients with other haematological malignancies are not at a statistically significant increased risk of all‐cause mortality compared to patients with digestive‐organ cancers but numbers remain too small to rule out small increases in risk, e.g. a 20% increase in risk (OR 1·2).

### The impact of anti‐cancer treatments

**Fig 2:**
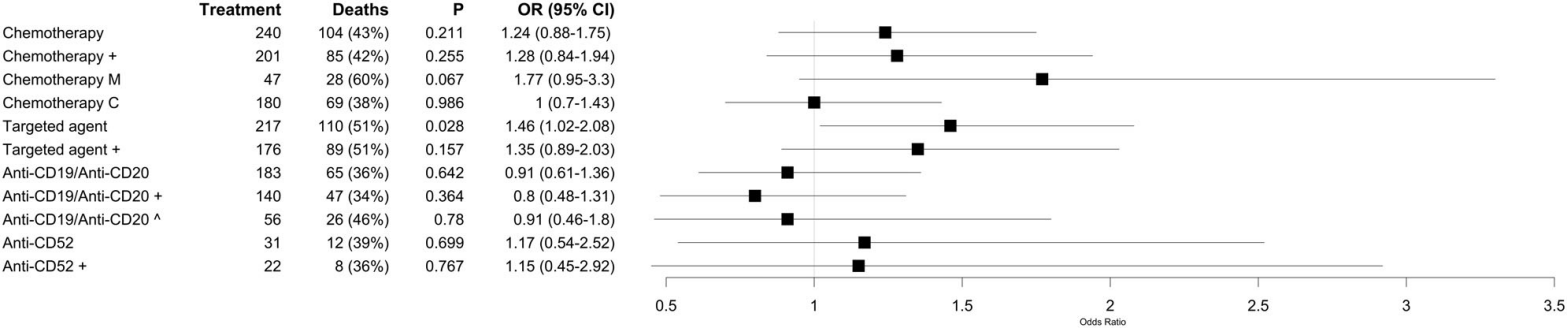
The impact of anti‐cancer treatments. The forest plot displays the odds ratio (OR) and 95% confidence interval (CI) calculated using a multivariate model testing for the effect of each treatment. Age, sex, comorbidity status and cancer type were included as covariates in all models. + indicates that line of treatment was also included as a covariable. ‘Chemotherapy M’ compared those on monotherapy chemotherapy to those not on any chemotherapy at all, whilst ‘Chemotherapy C’ compared those on combination chemotherapy to those not on any chemotherapy at all. Patients were considered on chemotherapy or targeted agents if these treatments were administered within four weeks of COVID‐19 diagnosis. Patients were considered on B‐ and T‐cell depletion if treated with these agents within two years of COVID‐19 diagnosis. We also tested the impact of recent (within four weeks of COVID‐19 diagnosis) B cell depletion (^). No results were generated to test the impact of radiotherapy and stem cell transplant because so few patients received these treatments (*n* = 9 and *n* = 8, respectively).

**Fig 3:**
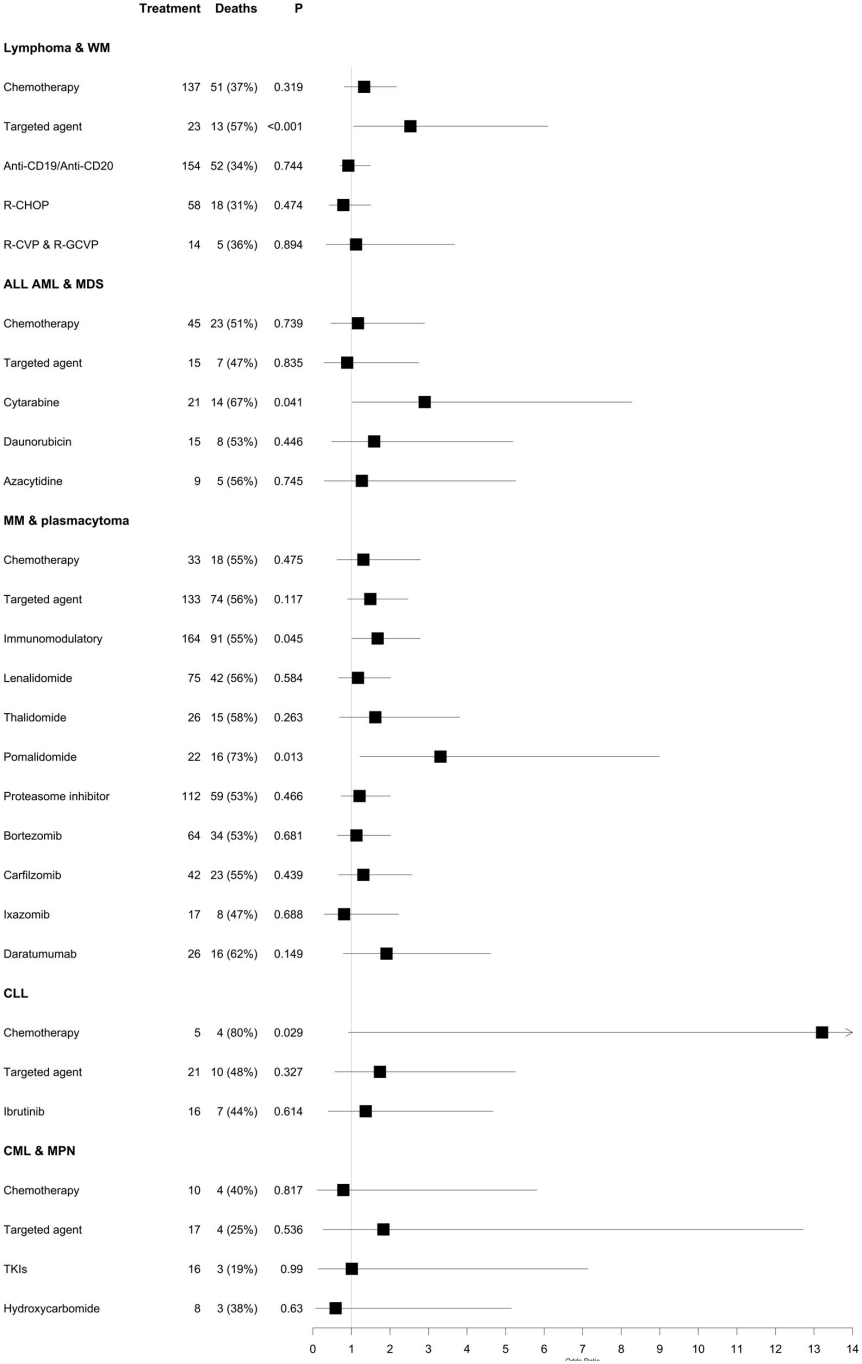
The impact of specific cancer treatments split by haematological cancer subtype. The forest plots display the odds ratio and 95% confidence interval calculated using a multivariate model testing for the effect of each treatment in each haematological cancer subtype indicated. The following paitent numbers were available for analysis in each subgroup: lymphoma & WM: *n* = 330, ALL, AML & MDS: *n* = 91, MM & plasmacytoma: *n* = 273, CLL: *n* = 80, CML & MPN: *n* = 35. Tyrosine kinase inhibitors (TKIs) included bosutinib, imatinib, ruxolitinib, nilotinib and dasatinib. In the analysis of CML & MPN 8/10 chemotherapies are hydroxycarbamide and 16/17 targeted agents are TKIs. The odds ratio calculated when testing the impact of chemotherapy in CLL patients is 13·5 [95% confidence interval (CI) = 0·92–189·53]. This result, whilst suggesting a possible high‐risk of chemotherapy in this group of patients, is not reliable, being based on only five patients. This result needs validation in larger sample sets. The smaller numbers in these subgroup analyses mean only results of large effect sizes will be detected. ALL, acute lymphoblastic leukaemia; AML, acute myeloid leukaemia; CLL, chronic lymphocytic leukaemia; CML, chronic myeloid leukaemia; MDS, myelodysplastic syndrome; MM, multiple myeloma; MPN, myeloproliferative neoplasms; TKI, tyrosine kinase inhibitor; WM, Waldenström macroglobulinaemia.

No significant associations were observed between all‐cause mortality and treatment with cytotoxic chemotherapy in the four weeks prior to COVID‐19 diagnosis (Fig 2; OR 1·12, 95% CI 0·85–1·6). We also did not observe a significant association between with anti‐CD52 or anti‐CD19/anti‐CD20 treatment within two years prior to COVID‐19 diagnosis [Fig 2, anti‐CD52: OR 1·17 (95% CI 0·54–2·52); anti‐CD19/CD20: OR 0·91 (95% CI 0·61–1·36)]. Whilst based on small numbers there was also no evidence of an adverse impact of anti‐CD19/anti‐CD20 within four weeks of COVID‐19 infection (Fig 2). Numbers were too small to test impact of very recent anti‐CD52 treatment. Treatment with targeted agents in the four weeks prior to COVID‐19 diagnosis was significantly associated with a higher risk of all‐cause mortality [OR = 1·46 (95% CI 1·02–2·09), *P* = 0·037; Fig 2], but this association did not remain after adjusting for line of treatment [OR = 1·35 (95% CI 0·9–2·05), *P* = 0·15; Fig 3].

The breakdown of cancer diagnoses and individual treatments tested are shown in Table II. Cytotoxic chemotherapy was not associated with all‐cause mortality in any subtype apart from CLL. for which only five CLL patients received chemotherapy, four of whom died [OR = 13·21 (95% CI 0·92–189·53), *P* = 0·029]. Targeted agents are a heterogeneous group and were not significantly associated with all‐cause death in any individual cancer type. The specific agents examined are provided in Table II. Only cytarabine in the analysis of AML, ALL and MDS was associated with a significantly increased risk of all‐cause mortality [OR = 2·90 (95% CI 1·02–8·28), *P* = 0·041].

**Table II:** A breakdown of the cancer types and cancer type‐specific treatments included in analyses restricted by haematological cancer subtype.

Subtype	*n*	Breakdown	Specific regimens investigated in this subtype
Lymphoma and WM	333	27 Hodgkin lymphomas; 55 follicular lymphomas; 78 non‐follicular lymphomas; 18 MTNKs; 96 non‐Hodgkin lymphomas; 16 WM; 45 other mature lymphoid haematopoietic cancers	Chemotherapy (*n* = 137), targeted agents (*n* = 23), B‐cell treatment (*n* = 154) R‐CHOP (*n* = 58), and R‐VCP or R‐GVCP (*n* = 14)
ALL, AML and MDS	97	17 ALL; 65 AML; 15 MDS	Chemotherapy (*n* = 45), azacytidine (*n* = 9), daunorubicin (*n* = 15), and targeted agents (*n* = 15), cytarabine (*n* = 23)
MM and plasmacytoma	275		Immunomodulatory drugs as a group and broken down into:75 receiving lenalidomide, 26 receiving thalidomide and 22 pomalidomide as part of their regimen [in 41 patients the exact immunomodulatory drug was unknown, chemotherapies (*n* = 33), targeted agents (*n* = 133), proteasome inhibitors as a group (*n* = 112) and, bortezomib (*n* = 64), ixazomib (*n* = 17) and carfilzomib (*n* = 42) separately, and daratumumab (*n* = 26)]
CLL	80		Chemotherapy *n* = 5, targeted therapies [*n* = 21, 16 of which were on the BTK inhibitor, ibrutinib]
CML and MPN	36	22 CML; 14 MPN. (Four of the MPN patients have myelofibrosis, three have polycythaemia vera and seven have essential thrombocythemia)	Targeted agents *n* = 17, 16 were taking tyrosine kinase inhibitors (including bosutinib, imatinib, nilotinib, dasatinib and ruxolitinib) or chemotherapy (*n* = 10 patients, 8 on hydroxycarbamide)

ALL, acute lymphoblastic leukaemia; AML, acute myeloid leukaemia; BTK, Bruton’s tyrosine kinase; CLL, chronic lymphocytic leukaemia; CML, chronic myeloid leukaemia; MDS, myelodysplastic syndrome; MM, multiple myeloma; MPN, myeloproliferative neoplasms; MTNK, mature T/NK‐cell lymphomas; R‐CHOP, rituximab, cyclophosphamide, doxorubicin, vincristine and prednisolone; R‐GVCP, Rituximab, gemcitabine, cyclophosphamide, vincristine and prednisolone; R‐VCP, rituximab, cyclophosphamide, vincristine and prednisolone; WM, Waldenström macroglobulinaemia.

In all, 164 patients with myeloma were actively treated with immunomodulatory drugs (IMIDs). Considering only patients with myeloma (*n* = 275), IMIDs were associated with increased risk of all‐cause death [OR = 1·68 (95% CI 1·01–2·79), *P* = 0·045]. We investigated the impact of individual IMIDs and identified that lenalidomide, the most commonly administered IMID (*n* = 75), was not associated with an increase in all‐cause death [OR = 1·17 (95% CI 0·67–2·02), *P* = 0·584] but pomalidomide was [OR = 3·31 (95% CI 1·22–8·99), *P* = 0·013]. Extent of prior treatment may be relevant to this association as, in the UK, pomalidomide is only reimbursed after three or more prior lines.

In the whole cohort, line of treatment was available for 644 patients (Table I). There were 146 not on treatment, 304 were receiving first‐line treatment and 194 were receiving second or higher lines of treatment. Compared to those not on treatment those receiving their second line of treatment or higher had a significantly increased risk of all‐cause mortality [OR = 1·68 (95% CI 1·07–2·64), *P* = 0·025].

### The impact of disease status

Haematological disease status was available for 527 patients. Compared to untreated asymptomatic patients (i.e. those on active surveillance only), patients with an indication to give the first treatment now and those who had been treated and had progressive or relapsed disease had higher risk of all‐cause death [OR = 2·45 (95% CI 1·09–5·5), *P* = 0·03 and OR = 3·21 (95% CI 1·68–6·14), *P* < 0·001 respectively]. Restricting the analysis by cancer type, only lymphoma and WM patients still showed a significant association [OR = 4·27 (95% CI 1·12–16·27), *P* = 0·034 and OR = 3·61 (95% CI 1·12–11·61), *P* = 0·031 respectively]. Asymptomatic untreated patients and those with ongoing responses to treatment had similar outcomes (Table I).

### The impact of laboratory parameters

Lymphopenia, observed in 467 patients, was associated with increased all‐cause death [OR = 1·61 (95% CI 1·14–2·27), *P* = 0·006]. Neutrophilia, observed in 105 patients, was also associated with increased risk of all‐cause death [OR = 1·83 (95% CI 1·18–2·83), *P* = 0·006]. Neutropenia and lymphocytosis were not significantly associated with all‐cause mortality in the whole cohort. Restricting the analysis by cancer type, lymphopenia was associated with an increased risk of mortality in MM and plasmacytoma [OR = 2·11 (95% CI 1·07–4·14), *P* = 0·029]. CRP levels >68 mg/l were observed in 300 patients, high CRP was associated with an increased risk of all‐cause death in the whole cohort [OR = 1·72 (95% CI 1·22–2·43), *P* = 0·002].

### Enrichment analyses

Comparison of the cancers registered to UKCCMP and  Office for National Statistics incidence data indicates overrepresentation of haematology cancer patients in UKCCMP compared to solid‐organ cancer patients [OR = 0·357 (95% CI 0·33–0·387), *P* = 3·21 × 10^−121^]. All subtypes of haematological malignancy are overrepresented in our dataset with the exception of MDS. Very similar results were obtained when UKCCMP data was compared to Haematological Malignancy Research Network data (Table SV).

## Discussion

These data confirm the high mortality risk of COVID‐19 in unvaccinated patients with haematological malignancy in a large cohort of UK patients. The highest mortality risk is in those with acute leukaemia and MDS. As in many series, age and comorbidities, neutrophilia, lymphopenia and high CRP are predictors of mortality. Mortality of patients admitted to ITU was also high at 68%. We have found that increasing line of treatment and disease requiring treatment or progressing after treatment are also both risk factors in this cohort, the latter of particular relevance when weighing the decision to give treatment.

In this cohort of patients, treatment with cytotoxic chemotherapy was not associated with increased risk of mortality, although a relatively small effect size cannot be excluded. We excluded patients treated with curative intent more than 12 months previously, which may explain the difference in effect of cytotoxic chemotherapy reported in other studies.^9^, ^10^ The lack of increased risk with anti‐CD20 B‐cell depleting treatments in this unvaccinated cohort is of particular interest given the concerns that have been expressed about such agents.

Given the small numbers of patients available for analysis when considering other individual treatments this study was only powered to detect large effect sizes and cannot rule out modest effects for treatments that we did not identify statistically significant results for. The significance of the increased mortality risk associated with cytarabine in patients with acute leukaemia and MDS, and with pomalidomide in myeloma is uncertain especially given the place of pomalidomide treatment in later lines of myeloma therapy and the relatively high non‐COVID‐19 mortality induction therapy for acute leukaemia. Importantly the patients in this cohort had not received COVID‐19 vaccination. The clinical efficacy of vaccines in patients with haematological cancers is uncertain and a key area of ongoing research.^21^

The strengths of these data include the large size of the cohort, thorough curation of individual patient characteristics and the treatment of patients within one national healthcare system. An important limitation of the data, as with other similar datasets, is the uncertain number of undiagnosed community cases during the early stages of the pandemic because of lack of availability of community testing in this period, as well as the usual limitations of observational data. Another limitation is the small numbers of patients treated with stem cell transplants, radiotherapy or anti‐CD52 (T‐cell depletion). This study is unable to provide useful information as to the impact of these treatments in patients with COVID‐19. Some covariates were also missing for many patients, making it impossible to adjust for them without compromising sample size. Multiple tests have been applied in this analysis and only the results of the impact of age and comorbidities and the enrichment analysis showing overrepresentation of patients with haematological malignancies in this cohort remain statistically significant upon applying a strict Bonferroni correction for multiple testing.

This dataset therefore confirms the high mortality of patients with SARS‐CoV‐2 in the UK population with haematological malignancy and identifies those with acute leukaemia and MDS as a group at particular risk. Our analysis emphasises the importance of disease control as a factor predicting mortality of as much importance as the possible risks of cytotoxic treatment or anti‐CD20 treatments.

## Funding information

The work was supported by University of Birmingham, University of Oxford, Blood Cancer UK (#20011). The funders of the study had no role in study design, data collection, data analysis, data interpretation, or writing of the report. This research was also supported by the National Institute for Health Research (NIHR) infrastructure at Leeds.

## Conflicts of interests

The authors have no conflict interests to declare.

## Ethics statement

The UKCCMP was classified by the NHS Health Research Authority as a Public Health Surveillance project that did not require further ethical review or approval by the HRA (letter available at www.UKcoronaviruscancermonitoring.com). All data were de‐identified at source. Only anonymised data wwere held on the project's REDCap database. Each participating centre provided a Centre Activation Form which was taken as confirmation that all required local approvals were in place (including Caldicott approval where deemed necessary by each Centre).
