# Supplementary material for: Key findings from the UKCCMP cohort of 877 patients with haematological malignancy and COVID‐19: disease control as an important factor relative to recent chemotherapy or anti‐CD20 therapy
Source: Br J Haematol. 2021 Nov 10;196(4):892–901. doi: 10.1111/bjh.17937 (PMC8652610; doi:10.1111/bjh.17937)
Supplement: Supplementary file 1 [file BJH-196-892-s001.docx]

## Supplementary Appendix

**Authors in the UKCCMP team and their affiliations**

| ***Author*** | ***Affiliation*** |
| --- | --- |
| Angelos Angelakas | University Hospitals of Morecambe Bay NHS Foundation Trust |
| Avinash Aujayeb | Northumbria Healthcare NHS Foundation Trust |
| Mark Baxter | NHS Tayside |
| James Best | Royal Shrewsbury Hospital |
| Madhumita Bhattacharyya | Royal Berkshire Hospital |
| Ruth Board | Lancashire Teaching Hospitals NHS Foundation Trust |
| Rachel Bolton | East Lancashire Hospitals NHS Trust |
| Joe Browning | Buckinghamshire Healthcare NHS Trust |
| Emma Cattell | Musgrove Park Hospital |
| Julia Chackathayil | St George’s University Hospital |
| Joseph Chacko | Royal Bournemouth Hospital |
| Lucia Chen | Oxford University Hospitals NHS Foundation Trust |
| Vinton WT Cheng | Leeds Institute of Medical Research, University of Leeds​, Leeds, United Kingdom |
| Ellen Copson | University Hospital Southampton NHS FT |
| Jennifer Davies | Buckinghamshire Healthcare NHS Trust |
| Sarah Derby | Glasgow, Beatson West of Scotland Cancer Centre |
| Louise Devereaux | Wigan, Wrightington and Leigh |
| Elissa Dhillon | Oxford University Hospitals NHS Foundation Trust |
| Nikolaos Diamantis | Barts Health NHS Trust |
| David Dutton | Great Western Hospitals |
| Shawn Ellis | Royal Berkshire Hospital |
| Laura Feeney | Northern Ireland Cancer Centre, Belfast |
| Mathew Fittal | Royal Marsden NHS Foundation Trust |
| Aisha Ghaus | Glasgow, Beatson West of Scotland Cancer Centre |
| Paul Greaves | Barking, Havering and Redbridge University Hospitals NHS Trust |
| Simon Grumett | Royal Wolverhampton Hospital NHS Trust |
| Madeleine Hewish | Ashford and St Peters |
| Michaela Hill | George Eliot Hospital NHS Trust |
| Laura Horsley | The Christie NHS Foundation trust |
| Zoe Hudson | University Hospitals Bristol and Weston |
| Daniel J Hughes | Department of Cancer Imaging, School of Biomedical Engineering and Imaging Sciences, King's College London, London, UK |
| Sangray Kathirgamakarthigeyan | Nottingham University Hospitals NHS Trust |
| Bartlomiej Kurec | Worcester Royal Hospital |
| Amy Kwan | Weston Park Hospital, Sheffield |
| Sapna Ladani | University Hospitals of Leicester NHS Trust |
| Alvin JX Lee | UCL Cancer Institute, University College London, London, UK |
| Rebecca Lee | Christie NHS Trust |
| Pauline Leonard | Barking, Havering and Redbridge University Hospitals NHS Trust |
| Shakeel Lowe | Hull and East Yorkshire NHS Trust |
| Samah Massalha | Betsi Cadwaladr University Health Board |
| Hayley Mckenzie | University Hospital Southampton NHS FT |
| Lucinda Melcher | North Middlesex University Hospital |
| Charlotte Moss | Translational Oncology and Urology Research, School of Cancer and Pharmaceutical Sciences, King’s College London, London, UK |
| Daniel Muller | Hampshire Hospitals NSHFT |
| Nirupa Murugaesu | Chelsea and Westminster |
| Jillian Noble | Croydon University Hospital |
| Roderick Oakes | North Cumbria Integrated Care NHS Foundation Trust |
| Anna C Olsson-Brown | The Clatterbridge Cancer Centre, Wirral, UK and The University of Liverpool, Liverpool, UK |
| Annet Pillai | Hull and East Yorkshire NHS Trust |
| Ashley Poonking | Velindre Cancer Centre |
| Matthew Poynton | Oxford University Hospitals NHS Foundation Trust |
| Timothy Robinson | University Hospitals Bristol and Weston |
| Tom Roques | Norfolk and Norwich University Hospital (NNUH) |
| Michael Rowe | Royal Cornwall Hospital |
| Beth Russell | Translational Oncology and Urology Research, School of Cancer and Pharmaceutical Sciences, King’s College London, London, UK |
| Martin Scott-Brown | University Hospital Coventry and Warwickshire |
| Martin Scott-Brown | George Eliot Hospital NHS Trust |
| Christopher Scrase | Ipswich Hospital |
| Archana Sharma-Oates | Institute of Inflammation and Ageing, University of Birmingham, Birmingham, UK |
| Omar Sheikh | Nottingham University Hospitals NHS Trust |
| Fiona Smith | University Hospitals of Derby and Burton |
| Christina Thirlwell | Royal Devon and Exeter |
| Mieke Van Hemelrijck | Translational Oncology and Urology Research, School of Cancer and Pharmaceutical Sciences, King’s College London, London, UK |
| John Willan | Frimley Health NHS Foundation Trust |
| Roslin Zuha | Epsom and St Helier |


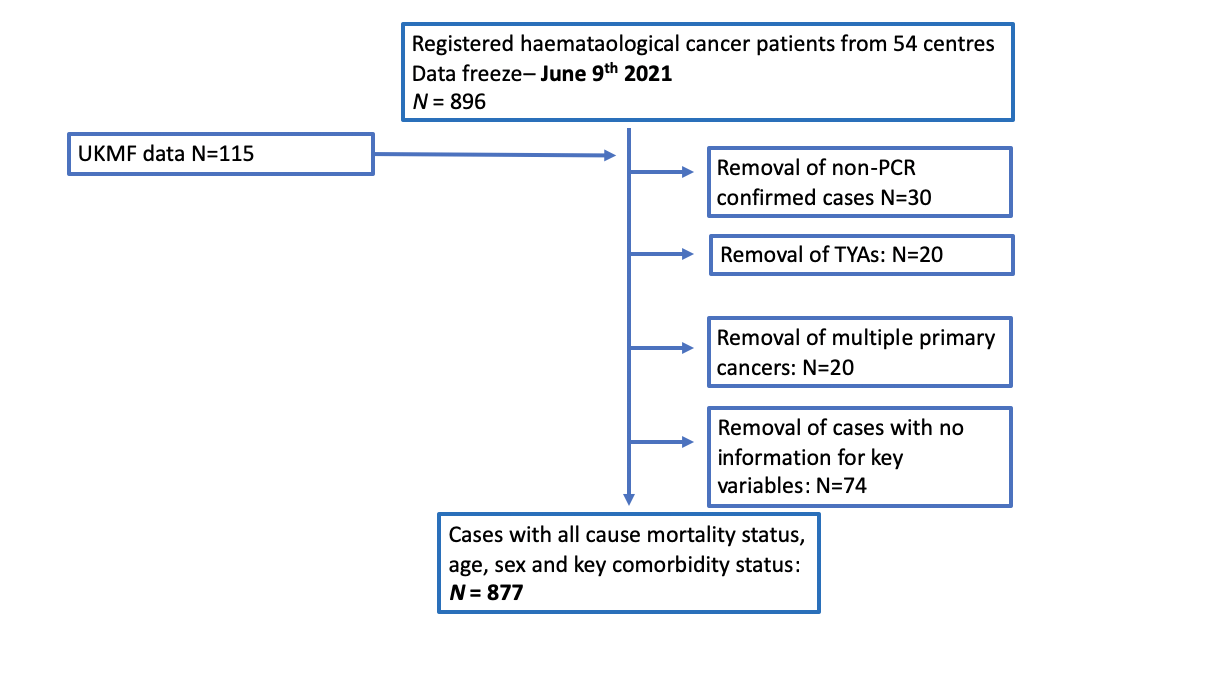


**S Figure I: CONSORT diagram for UKCCMP and UKMF patients included in the analysis**

*The number of patients available for analysis and the reasons for exclusions from the final dataset for analysis are provided.*

| ***Drug*** | ***Drug Class*** |
| --- | --- |
| ALEMTUZUMAB | TARGETED THERAPY AND T CELL DEPLETING |
| ANTI-CD38 | TARGETED THERAPY |
| ATEZOLIZUMAB | IMMUNOTHERAPY |
| AZACITIDINE | CHEMOTHERAPY |
| AZATHIOPRINE | IMMUNOSUPPRESION |
| BEXAROTENE, IFN ALPHA, PHOTOPHERESIS | OTHER THERAPY |
| BEAM PROTOCOL | CHEMOTHERAPY |
| BENDAMUSTINE | CHEMOTHERAPY AND T CELL DEPLETING |
| BENDAMUSTINE | CHEMOTHERAPY AND T CELL DEPLETING |
| BENTUZIMAB | TARGETED THERAPY |
| BISPHOSPHONATE | SUPPORTIVE TREATMENT |
| BLEOMYCIN | CHEMOTHERAPY |
| BLINOTUMUMAB | ANTI-CD19 – B-cell depleting |
| BORTEZOMIB | TARGETED THERAPY |
| BOSUTINIB | TARGETED THERAPY |
| BRENTUXIMAB | TARGETED THERAPY |
| BUDESONIDE | IMMUNOSUPPRESION |
| CARBOPLATIN | CHEMOTHERAPY |
| CARFILZOMIB | TARGETED THERAPY |
| CHLORAMBUCIL | CHEMOTHERAPY |
| CHOP | CHEMOTHERAPY |
| CICLOSPORIN | IMMUNOSUPPRESION |
| CISPLATIN | CHEMOTHERAPY |
| CLADRIBINE | T CELL DEPLETING |
| CPX-351 | CHEMOTHERAPY |
| CYCLOPHOSPHAMIDE | CHEMOTHERAPY |
| CYTARABINE | CHEMOTHERAPY |
| DACARBAZINE | CHEMOTHERAPY |
| DARATUMUMAB | TARGETED THERAPY |
| DASATINIB | TARGETED THERAPY |
| DAUNORUBICIN | CHEMOTHERAPY |
| DEXAMETHASONE | STEROID |
| DOXORUBICIN | CHEMOTHERAPY |
| ETOPOSIDE | CHEMOTHERAPY |
| FLUDARABINE BUSULPHAN | CHEMOTHERAPY |
| GEMCITABINE | CHEMOTHERAPY |
| GEN BISPECIFIC ANTIBODY | ANTI CD-20/3 – B cell depleting |
| HDAC | CHEMOTHERAPY |
| HYDROXYCARBIMIDE | CHEMOTHERAPY |
| IBRUTINIB | TARGETED THERAPY |
| IDARUBICIN | CHEMOTHERAPY |
| [IFOSFAMIDE](https://www.macmillan.org.uk/cancer-information-and-support/treatments-and-drugs/ifosfamide) | CHEMOTHERAPY |
| IMATINIB | TARGETED THERAPY |
| IMMUNOMODULATORY | IMMUNOMODULATORY |
| INOTUZUMAB | TARGETED THERAPY |
| ISATUXIMAB | TARGETED THERAPY |
| IXABEPILONE | TARGETED THERAPY |
| IXAZOMIB | TARGETED THERAPY |
| LD56 | CHEMOTHERAPY |
| LENOLIDAMIDE | IMMUNOMODULATORY |
| LIPOSOMAL DAUNORUBICIN AND CYTARABINE | CHEMOTHERAPY |
| MATRIX | CHEMOTHERAPY |
| MDM2 INHIBITOR | CHEMOTHERAPY |
| MECAPTOPURINE | CHEMOTHERAPY |
| MELPHALAN | CHEMOTHERAPY |
| MELPHCON | CHEMOTHERAPY |
| METHOTREXATE | CHEMOTHERAPY |
| METHYLPREDNISLONE STEROIDS | STEROID |
| MIDOSTAURIN | TARGETED THERAPY |
| MMF | IMMUNOSUPPRESION |
| MYLOTARG | CHEMOTHERAPY |
| NILOTINIB | TARGETED THERAPY |
| NIVOLUMAB | IMMUNOTHERAPY |
| OBINUTUZUMAB | ANTI CD-20 – targeted B cell depleting |
| ON TKI MAINTENANCE | TARGETED THERAPY |
| PANOBINOSTAT | TARGETED THERAPY |
| PEG-ASPARAGINASE | CHEMOTHERAPY |
| PENTOSTATIN MONOTHERAPY | CHEMOTHERAPY AND T CELL DEPLETING |
| PIRTOBRUTINIB | TARGETED THERAPY |
| POLATUZUMAB | TARGETED THERAPY |
| POMALIDOMIDE | IMMUNOMODULATORY |
| PONATINIB | TARGETED THERAPY |
| PREDNISOLONE | STEROID |
| PROCARBAZINE | CHEMOTHERAPY |
| PROTEASOME INHIBITOR | TARGETED THERAPY |
| RITUXIMAB | ANTI CD-20 – B cell depleting |
| RO7082859 | TARGETED THERAPY AND ANTI CD-20/3 – B cell depleting |
| RUXOLITINIB | TARGETED THERAPY |
| TACROLIMUS | IMMUNOSUPPRESION |
| TGR-1202 | TARGETED THERAPY |
| THALIDOMIDE | IMMUNOMODULATORY |
| THIOTEPA | CHEMOTHERAPY |
| TLA ATG | CHEMOTHERAPY, STEM CELL TRANSPLANT AND T CELL DEPLETING |
| TRETINOIN ARSENIC | TARGETED THERAPY |
| UBLITUXIMAB | ANTI CD-20 – B cell depleting |
| UKALL 14 CONSOLIDATION | CHEMOTHERAPY |
| UMBRALISIB | TARGETED THERAPY |
| VENETOCLAX | TARGETED THERAPY |
| VINBLASTINE | CHEMOTHERAPY |
| VINCRISTINE | CHEMOTHERAPY |
| ZOLEDRONIC ACID | SUPPORTIVE TREATMENT |

***Table SI: Matching drug names to treatment categories.***

*This table shows how the drugs patients were on in the four weeks before COVID-19 was diagnosed were grouped into treatment categories for analysis.*

|  | Overall count (N) in all-cause death test | Number of all cause deaths | P value | OR  (95% CI) | Overall count (N) in COVID-19 specific death test | Number of COVID-19 specific deaths (N) | P value | OR  (95% CI) |
| --- | --- | --- | --- | --- | --- | --- | --- | --- |
| Whole cohort | 877 | 384 (44%) | - | - | 787 | 294 (37%) | - | - |
| Median age (range)* | 71 (25-98) | 74 (25-95) | < 0.001 | 1.04  (1.03-1.06) | 70 (25-98) | 73 (25-95) | < 0.001 | 1.04  (1.03-1.05) |
| Aged 65 and older | 575 (66%) | 293 (51%) | < 0.001 | 2.41  (1.79-3.24) | 504 (64%) | 222 (44%) | < 0.001 | 2.31  (1.68-3.18) |
| Male | 547 (62%) | 309 (63%) | 0.832 | 0.97  (0.74-1.28) | 491 (62%) | 182 (37%) | 0.829 | 0.97  (0.72-1.3) |
| Has key comorbidity | 431 (49%) | 201 (52%) | 0.095 | 1.26  (0.96-1.64) | 408 (52%) | 178 (44%) | < 0.001 | 1.75  (1.31-2.35) |
| Number of key comorbidities*^ | 346: 217: 118:49:11:4 | 126:91:61:24:6:1 | 0.006 | 1.21  (1.06-1.4) | 334:210:113:43:10:4 | 114:84:56:18:5:1 | 0.019 | 1.19  (1.03-1.37) |
| Black, Asian and minority ethnicities (BAME) | 141 BAME, 490 white, 246 no data | 58 (41%) BAME  210 (43%) white | 0.715 | 0.93  (0.64-1.36) | 131 BAME, 439 white, 217 no data. | 48 (37%) BAME, 280 (36%) white | 0.93 | 1.02  (0.68-1.53) |

***Table SII Impact of Age, Sex, Comorbidities and Ethnicity on all-cause and COVID-19 specific death.***

*Results of univariate analyses exploring age, sex, comorbidities and ethnicity are presented.(*) indicates continuous variables, (^) indicates a quantitative variable with levels 0-5. For the all-cause death outcome, mean follow-up period was 24 days for alive patients and 12 days for deceased patients. For the COVID-specific death outcome the mean follow-up period for alive patients was 24 days and 11 days for deceased patients.*

|  | Overall count | All cause death | P value | OR (95% CI) | COVID-19 specific death | Provided oxygen | Provided ITU |
| --- | --- | --- | --- | --- | --- | --- | --- |
| Chronic kidney disease | 83 (12%) | 41 (14%) | 0.12 | 1.44 (0.91- 2.28) | 35 (13%) | 51 (14%) | 12 (17%) |
| Chronic obstructive pulmonary disease | 37 (5%) | 17 (6%) | 0.57 | 1.21 (0.62- 2.36) | 15 (6%) | 24 (7%) | 2 (3%) |
| Cardiovascular disease | 104 (15%) | 54 (19%) | 0.02 | 1.65 (1.08-2.5) | 49 (18%) | 69 (19%) | 6 (9%) |
| Diabetes mellitus | 132 (19%) | 59 (20%) | 0.402 | 1.18 (0.8-1.72) | 51 (19%) | 80 (22%) | 11 (16%) |
| Hypertension | 224 (32%) | 106 (36%) | 0.031 | 1.42 (1.03-1.96) | 98 (37%) | 133 (37%) | 21 (30%) |
| Vascular | 41 (6%) | 21 (7%) | 0.194 | 1.52 (0.81-2.86) | 19 (7%) | 27 (7%) | 3 (4%) |

***Table SIII Impact of individual comorbidities on all-cause mortality, COVID-19 specific mortality, whether patient was provided with oxygen and whether patient was provided with ITU care.***

*Row percentages indicate the number of patients with the comorbidity out of the dataset of 877 patients. Obesity was not included in multivariate analyses as only 446/877 had a BMI measurement. 107 were considered obese and of these 47% died of all cause death. There was no significant association between all cause death and obesity observed (OR=1.26, 95%CI 0.82-1.95, P=0.297).*

|  | **Comparison to all other subgroups** | | | | **Comparison to lymphoma & WM** | | | |
| --- | --- | --- | --- | --- | --- | --- | --- | --- |
|  | Overall count (N) | Number of all cause deaths | P value | OR  (95% CI) | Overall count (N) | Number of all cause deaths | P value | OR  (95% CI) |
| ALL, AML & MDS | 97 | 49 (51%) | 0.018 | 1.71  (1.09-2.69) | 97 | 49 (51%) | 0.002 | 2.12  (1.31-3.44) |
| CLL | 80 | 33 (43%) | 0.449 | 0.83  (0.51-1.35) | 77 | 37 (48%) | 0.575 | 1.16  (0.69-1.93) |
| MPN & CML | 36 | 13 (33%) | 0.340 | 0.71  (0.35-1.44) | 36 | 11 (31%) | 0.691 | 0.86  (0.4-1.84) |
| Lymphomas & WM | 333 | 119 (36%) | 0.003 | 0.64  (0.48-0.86) | - | - | - | - |
| Myeloma & plasmacytoma | 275 | 141 (51%) | 0.086 | 1.3  (0.96-1.76) | 275 | 141 (51%) | 0.008 | 1.58  (1.13-2.23) |
| Other/unspecified | 56 | 29 (52%) | 0.148 | 1.51  (0.86-2.66) | 56 | 29 (52%) | 0.027 | 1.95  (1.08-3.51) |

***Table SIV Impact of haematological cancer type on all-cause mortality***

*The impact of cancer subtype was investigated by i) comparing those with a single subtype to all other patients in the dataset and ii) comparing those with a single subtype to those with lymphoma and WM.*

*Results presented are from multivariate models including age, sex and key comorbidity status. For the comparison to all other subgroups each cancer type was compared to the rest of the cohort to generate the per cancer type statistics. In order to confirm that there was no difference in risk for patients with MPN and patients with CML we evaluated this group of cancers together as well as separately. Results for the combined analysis of MPN and CML patients is shown in the table. Results were similar CML only: OR =0.66 (95% CI 0.25,1.76), P=0.395 and MPN only: OR=0.63 (95% CI 0.2,1.93), P=0.409. The mean follow-up period for alive patients was 24 days whilst for deceased patients mean follow up was 12 days. For the multivariate analysis using lymphoma & WM as the comparator group, the overall p-value=0.007 indicating that patients with lymphoma and WM have a significantly better outcome than the other cancer types.*

|  | Comparator Value (%) | UKCCMP value (%) | P value | OR (95% CI) |
| --- | --- | --- | --- | --- |
| **UKCCMP versus Haematological Malignancy Research Network data** | | | | |
| Lymphoma & WM | 13758 (31.1%) | 368 (42.4%) | 1.41x10^-09^ | 0.652(0.568-0.75) |
| MM & plasmacytoma | 4800 (11.3%) | 187 (21.5%) | 1.88x10^-17^ | 0.465(0.394-0.551) |
| ALL, AML & MDS | 5922 (14%) | 101 (14.3%) | 0.766 | 0.974(0.802-1.19) |
| ALL & AML | 3071 (7.2%) | 98 (11.3%) | 2.27x10^-05^ | 0.613(0.495-0.767) |
| MDS | 2851 (6.7%) | 26 (3%) | 2.4x10^-06^ | 2.334(1.578-3.601) |
| CLL | 4680 (11%) | 85 (9.8%) | 0.273 | 1.143(0.91-1.45) |
| MPN & CML | 4869 (11.5%) | 36 (4%) | 1.77x10^-14^ | 3.09(2.2-4.47) |
| Other/unspecified | 1440 (3.4%) | 63 (7.3%) | 5.33x10^-08^ | 0.449(0.345-0.593) |
| **UKCCMP versus Office for National Statistics Cancer Registrations data** | | | | |
| All haematology | 37229 (12.5%) | 868 (28.6%) | 3.21x10^-121^ | 0.357(0.33-0.387) |
| Lymphoma & WM | 12729 (4.3%) | 368 (12.1%) | 1.1x10^-68^ | 0.324(0.29-0.363) |
| MM & plasmacytoma | 5033 (1.7%) | 187 (6.2%) | 9.1x10^-49^ | 0.262(0.225-0.306) |
| MDS | 2377 (0.8%) | 26 (0.9%) | 0.681 | 0.932(0.633-1.431) |
| Lymphoid leukaemia | 3785 (1.3%) | 107 (3.5%) | 1.07x10^-19^ | 0.352(0.29-0.433) |
| Myeloid leukaemia | 3939 (1.3%) | 111 (3.7%) | 2.77x10^-20^ | 0.353(0.3-0.43) |

***Table S V Comparison of the incidence of haematological cancers in the UKCCMP dataset to incidence data from the Haematological Malignancy Research Network & Office for National Statistics.***

*P values were generated using Fishers exact test (two sided). All cancers that were PCR confirmed were used in this analysis, regardless of complete data otherwise. Multiple myeloma cases from UKMF (N=113) were not included in the analysis. 2017* Office for National Statistics *data from England was analysed.* Haematological Malignancy Research Network *data was obtained in March 2021.  UKCCMP data was collected between 1st March 2020 and 9th June 2021.*
